# Artificial Intelligence to Improve Antibiotic Prescribing: A Systematic Review

**DOI:** 10.3390/antibiotics12081293

**Published:** 2023-08-06

**Authors:** Doaa Amin, Nathaly Garzόn-Orjuela, Agustin Garcia Pereira, Sana Parveen, Heike Vornhagen, Akke Vellinga

**Affiliations:** 1 School of Public Health, Physiotherapy & Sports Science, University College Dublin, Belfield, Dublin 4, Dublin, Ireland; nathaly.garzonorjuela@ucdconnect.ie (N.G.-O.); sana.parveen@ucd.ie (S.P.); akke.vellinga@ucd.ie (A.V.); 2 Insight Centre for Data Analytics, University of Galway, H91 AEX4 Galway, Ireland; agustin.garciapereira@insight-centre.org (A.G.P.); heike.vornhagen@insight-centre.org (H.V.)

**Keywords:** antibiotic prescribing, antibiotic resistance, artificial intelligence (AI), machine learning (ML), prediction, human patients

## Abstract

*Introduction:* The use of antibiotics leads to antibiotic resistance (ABR). Different methods have been used to predict and control ABR. In recent years, artificial intelligence (AI) has been explored to improve antibiotic (AB) prescribing, and thereby control and reduce ABR. This review explores whether the use of AI can improve antibiotic prescribing for human patients. *Methods:* Observational studies that use AI to improve antibiotic prescribing were retrieved for this review. There were no restrictions on the time, setting or language. References of the included studies were checked for additional eligible studies. Two independent authors screened the studies for inclusion and assessed the risk of bias of the included studies using the National Institute of Health (NIH) Quality Assessment Tool for observational cohort studies. *Results:* Out of 3692 records, fifteen studies were eligible for full-text screening. Five studies were included in this review, and a narrative synthesis was carried out to assess their findings. All of the studies used supervised machine learning (ML) models as a subfield of AI, such as logistic regression, random forest, gradient boosting decision trees, support vector machines and K-nearest neighbours. Each study showed a positive contribution of ML in improving antibiotic prescribing, either by reducing antibiotic prescriptions or predicting inappropriate prescriptions. However, none of the studies reported the engagement of AB prescribers in developing their ML models, nor their feedback on the user-friendliness and reliability of the models in different healthcare settings. *Conclusion:* The use of ML methods may improve antibiotic prescribing in both primary and secondary settings. None of the studies evaluated the implementation process of their models in clinical practices. *Prospero Registration:* (CRD42022329049).

## 1. Introduction

Between 2000 and 2010, global human antibiotic consumption increased by 35%, with a noticeable increase in the use of ‘last resort’ antibiotics, especially in middle-income countries [1]. Antibiotic resistance (ABR) is defined as the ability of bacteria to grow and adapt in the presence of antibiotics [2,3,4]. There is a direct association between antibiotic consumption and the emergence of antibiotic resistance (ABR) [5,6,7]. Inappropriate and excessive prescribing of antibiotics contributes to the spread of ABR [6,8,9]. ABR is associated with morbidities, hospital admissions, increased cost of healthcare, and treatment failures [10,11,12], and it has been listed as one of the top ten threats by the World Health Organization (WHO) [13]. In 2019, ABR was associated with 4.95 million deaths globally, of which 1.27 million deaths were directly attributable to ABR, with the highest number of deaths in Western Sub-Saharan Africa and the lowest number of deaths in Australasia [14]. Without concerted action, it is estimated that by 2050, worldwide, ABR will result in 300 million deaths, reduce GDP by 2.5–3% and losses of USD 60–100 trillion [15].

Different methods have been used to improve antibiotic prescribing, such as antibiotic stewardship programs (ASPs) (defined as set of interventions that promote the responsible usage of antibiotics) [16,17], and clinical decision support systems (CDSSs) (which are a source of patient-related recommendations and assessments for clinicians to help in their decision making [18]. These methods target a change in behaviour [19,20]. In addition, ASPs generally have a short-term effect [21], and need continued effort to obtain relatively small reductions in prescribing [22]. CDSSs are computer-based systems that are not always easy to integrate into the patient management systems, or in the workflow of the clinician [23].

Artificial intelligence (AI) is the ability of a machine, such as a computer, to “independently replicate intellectual processes typical of human cognition in deciding on an action in response to its perceived environment to achieve a predetermined goal” [24]. Machine learning (ML) is a subfield of AI [24,25], where machines are able to learn from data and improve their analyses by using computational algorithms [26,27]. Types of ML algorithms are supervised learning, unsupervised learning and reinforcement learning [24,28,29]. Supervised learning algorithms are those that perform prediction, and some of these algorithms perform classification based on previous data examples [28]. Examples of supervised learning algorithms are logistic regression [30], naïve bayes, support vector machine (SVM), decision trees [31], random forests [32], artificial neural networks (ANNs) [33] and gradient boosting [34]. On the other hand, unsupervised learning algorithms have the ability to explore patterns in data [28], for example, principal component analysis (PCA). Reinforcement learning algorithms are concerned with how an agent (i.e., algorithm) takes action in a space so that it can maximise a cumulative reward [31]. AI and ML, and other terms, such as data science, are often used interchangeably [35]. 

ML has been utilised in solving medical challenges [36], such as predicting cancer types, medical imaging, wearable sensors [37], healthcare monitoring [38], drug development, disease diagnostics, analysis of health plans, digital consultations and medical and surgical personalised treatments [39]. Recently, ML methods have been explored as means to guide the rational use of antibiotics, explore suitable antibiotic combinations or identify new antibiotic peptides (ABPs) [33]. No previous reviews were identified on the use of AI to improve antibiotic prescribing. The aim of this review was to explore the use of AI to improve antibiotic prescriptions for human patients.

## 2. Materials and Methods

The protocol for this review was registered in the PROSPERO database (CRD42022329049), and the “Preferred Reporting Items for Systematic Reviews and Meta-Analyses (PRISMA)” guideline [40] was used to design and report findings.

### 2.1. Eligibility Criteria

- Participants and setting: Participants were human patients without restrictions on their characteristics (i.e., gender, age, weight, morbidities). No restrictions were applied for setting (i.e., primary, secondary, or tertiary care), timing of publication or language of the studies included. The “no restriction” on participants′ inclusion, setting or language was applied to ensure that all relevant studies were retrieved;
- Study design: the studies included in this review were cohort studies or any observational study that examined the potential or actual use of AI, machine learning or data analytics to improve antibiotic prescribing or consumption in human patients;
- Outcome measures:
  ○ The relative reduction of antibiotic prescriptions (primary outcome);
  ○ The prediction of inappropriate antibiotics prescriptions;
  ○ The relative reduction in re-consultations of patients, irrespective of the reason for re-consultation (infection recurrence or worsening of patient’s condition).

### 2.2. Data Sources and Search Strategy

- Data sources: the search strategy was applied to Scopus, OVID, ScienceDirect, EMBASE, Web of Science, IEEE Xplore and the Association for Computing Machinery (ACM);
- Search strategy: the search strategy, which can be accessed in [41], was based on three different concepts: (1) artificial intelligence, (2) prescriptions, and (3) population, and was customised to each database, depending on the special filters of these databases. The search for studies stopped in April 2022, and the snowball search (i.e., screening the references of the included studies for additional eligible studies) was conducted in August 2022.

### 2.3. Study Records

#### 2.3.1. Study Selection

Two independent authors (DA, NG, AGP, SP, HV) scanned titles and abstracts of the studies to assess eligibility. Manuscripts of eligible studies were retrieved for full-text screening, and their references were checked for additional studies (i.e., snowball search). Two independent authors (DA, NG, SP) screened the full text of the retrieved studies. Whenever a conflict occurred, a third reviewer (AV) was involved in the resolution.

#### 2.3.2. Data Extraction and Management

A customised data extraction sheet was developed, where the main sections were:

- Study information, for example, publication type, country, name of publication outlet, authors, year of publication, title, aim of study and funding source;
- Population, for example, total number of participants, cohorts, sample size calculation, methods of recruitment, age group, gender, race/ethnicity, illness, comorbidities and inclusion/exclusion criteria of patients;
- Methods and setting, for example, design of the study, data source, setting, start and end dates, machine learning methods used, training and test sets, predictors, data overfitting, valuation and validation of performance and handling of missing data;
- Outcomes, such as outcome(s) name(s) and definition and, if applicable, unit of measurement and scales;
- Results, limitations and key conclusions.

One author (DA) extracted the data, which was reviewed by a second author (NG, SP). 

#### 2.3.3. Assessment of Risk of Bias

The National Institute of Health (NIH) Quality Assessment Tool for Observational Cohort and Cross-Sectional Studies was used to assess the quality of included studies [42]. The assessment was carried out by three authors (DA, NG, SP), and conflicts were resolved by discussion.

#### 2.3.4. Data Synthesis

Due to the small number of included studies, it was not feasible to assess the heterogeneity using I^2^ statistics. Instead, a narrative synthesis was adopted to analyse the studies in this review.

## 3. Results

**Figure 1:**
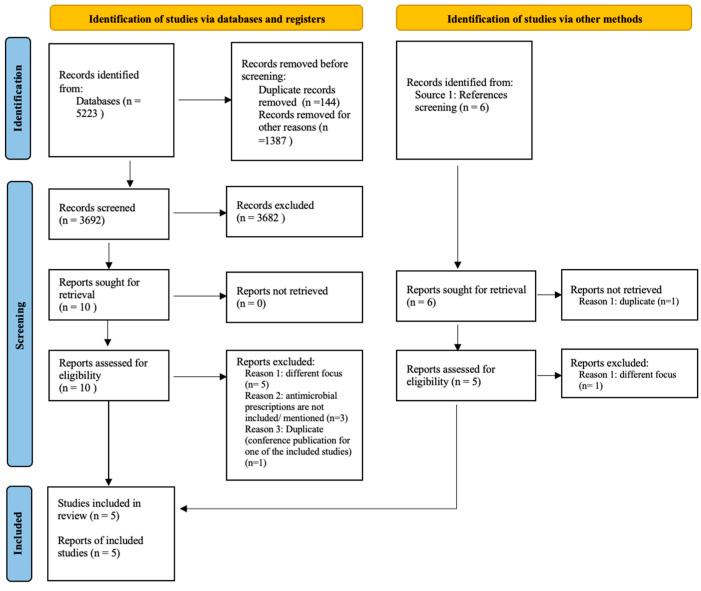
Prisma flow diagram [48]: studies selection process.

A total of 5223 citations were retrieved, and after removing the duplicates and irrelevant records (for example, posters, book chapters, and abstracts), 3692 records were included for screening. A total of fifteen studies were eligible for full-text screening; ten studies resulted from abstract screening, and five additional studies were identified from references screening (see Supplementary Materials Table S4 for reasons of exclusion in title and abstract screening). A final five studies [43,44,45,46,47] were included in the narrative synthesis and risk of bias assessment (see Figure 1). The characteristics of the included studies are available in Supplementary Materials Table S1, and the list of excluded studies is available in Supplementary Materials Table S2.

### 3.1. Included Studies

#### 3.1.1. Study Information and Population

Patients included were from different populations; female patients with UTIs (18–55 years old) [43], inpatients receiving an antibiotic [45], inpatients with UTIs [46] and children [44,47]. The children who were included in the Cambodian study had bloodstream infections (BSI) [47]. The population sizes ranged from 243 children with BSI [47] to 700,000 episodes of community-acquired urinary tract infections (UTIs), with 5,000,000 records of antibiotic purchases [46].

The five included studies [43,44,45,46,47] were in English and published between 2016 and 2022. The countries where the studies were conducted were the United States [43], South Korea [44], Canada [45], Israel [46] and Cambodia [47]. There was no information provided in any of the five studies [43,44,45,46,47] about sample size calculation, nor comorbidities or ethnicity.

#### 3.1.2. Methods and Settings

- Study design and setting: four studies [43,44,46,47] were retrospective, and one [45] was prospective (see Supplementary Materials Table S1). The setting in four studies [43,44,45,47] was secondary care and primary care in one study [46]. The data sources in four studies [43,44,45,47] were the electronic health records (EHRs) of the hospitals, while in a study by Yelin et al., the data of community- and retirement home-acquired urinary tract infections (UTIs) was obtained from Maccabi Healthcare Services (MHS), the second largest healthcare maintenance organisation in Israel [46]. All five studies [43,44,45,46,47] used supervised machine learning algorithms [28];
- Machine learning models in included studies:
  ○ Duration: the time frame of the data in the five included studies [43,44,45,46,47] ranged from 11 months to 10 years;
  ○ Predicted (outcome) variables: the primary outcome (relative reduction in antibiotic prescriptions) was reported in one study [43]; however, it was reported as a “proportion of recommendations for second-line antibiotics” (i.e., reduction in the use of second-line antibiotics) (see Table 1 and Supplementary Materials Table S1). The second outcome (prediction of inappropriate antibiotic prescriptions) was reported in five studies [43,44,45,46,47]; however, it was defined differently in each of them (Supplementary Materials Table S1). In a study by Kanjilal et al., it was reported as a “proportion of recommendations for inappropriate antibiotic therapy” [43], while in a study by Lee et al., a prediction of “antibiotic prescription errors” [44]. In a study by Beaudoin et al., it was reported as a prediction of “inappropriate prescriptions of piperacillin–tazobactam” [45]. The outcome in the study by Yelin et al. was a prediction of “mismatched treatments” (i.e., when the sample is resistant to the prescribed antibiotic) [46], and in a study by Oonsivilai et al., the outcome was a prediction of “susceptibility to antibiotics” [47]. None of the five studies [43,44,45,46,47] reported the third outcome (i.e., the relative reduction in re-consultations of patients);
  ○ Predictors: predictors in the studies included in this review were categorised as either “bedside” or “non-bedside” to make a distinction in the data a clinician had access to at diagnosis and prescription of the (empiric) treatment, and data that was only available after laboratory or other investigations [49]. Furthermore, predictors were divided into 10 groups: labs, antibiotics, demographics, geographical, temporal, socioeconomic conditions, gender-related, comorbidities, vital signs and medical history of patients (see Supplementary Materials Table S3). All groups of predictors used in the five studies [43,44,45,46,47] belonged to the “bedside” category, except for the lab′s group of predictors, which belonged to the “non-bedside” category. Since the clinical predictors are indicative in prescribing treatments, the inclusion in the development of ML models provides more accurate prediction compared to the clinicians who did not have this information at the time of prescribing.

Labs predictors were used in the five studies [43,44,45,46,47], while antibiotic-related predictors were used in four studies [43,44,46,47]. Demographic data were used in four studies [43,45,46,47], geographical predictors were used in three studies [43,45,46], and temporal predictors were used in two studies [46,47]. The study by Oonsivilai et al. was the only one that used socioeconomic-related predictors [47], and the study by Yelin et al. was the only one that included gender-related predictors (i.e., pregnancy) since its population was females with UTIs [46]. The comorbidities predictor was used in one study [43], as were vital signs predictors [45]. The medical history of patient-related predictors were reported in three studies [43,44,47] (Table 1). In addition, in a study by Kanjilal et al., there were two population-level predictors that belonged to both clinical and antibiotics groups of predictors [43].

○ Training and test datasets: A train/test split approach is used to ensure that the performance of an ML model is validated [50]. Three studies [43,46,47] reported that a train/test split approach was used, while two studies [44,45] did not report information about a train/test split. In the study by Kanjilal et al., the train/test split consisted of a training dataset of 10,053 patients versus a test dataset of 3941 patients [43], while in the study by Yelin et al., the data was divided in a way that all data collected from 1 July 2007 to 30 June 2016 was treated as a training set, and data collected from 1 July 2016 to 30 June 2017 was treated as test set [46]. In the remaining study, Oonsivilai et al. made an 80% versus 20% train/test split [47];
○ Machine learning models: All models [43,44,45,46,47] belong to the supervised machine learning models [28]. Three studies [43,46,47] used logistic regression and decision trees, while random forest models were used in two studies [43,47]. Gradient boosting decision trees (GBDTs) were used in [46,47], in addition to linear, radial and polynomial support vector machines (SVMs) and K-nearest neighbours (KNNs) in [47]. Two studies [44,45] used different models: an advanced rule-based deep neural network (ARDNN) and a supervised learning module (i.e., temporal induction of classification models (TIM));
○ Method to avoid data overfitting: Data overfitting occurs when an ML model perfectly fits training data but fails to generalise to test data [51], for which regularisation can be a solution [52]. Regularisation adds a penalty to the algorithm’s smoothness or complexity to avoid overfitting and improve the generalisation of the algorithm [53]. In the studies by Kanjilal et al. [43] and Oonsivilai et al. [47], regularisation was used to avoid data overfitting. In the study by Beaudoin et al. [45], the “J-measure”, which is defined as a measure of the goodness-of-fit of a rule [54], was used to reduce overfitting, and in the study by Yelin et al., the model’s performance on the test set was contrasted with the training set to identify data overfitting [46]. The study by Lee et al. did not report on data overfitting [44];
○ Handling missing data: Two studies [43,45] did not report how missing data were handled. In the study by Lee et al., 2.45% of height and weight missing data were predicted, other empty data records were deleted, and data outliers were treated as missing values [44]. In the study by Yelin et al., the missing data for antibiotic resistance was treated as “not available” and eventually dropped from the models [46]. In the study by Oonsivilai et al., missing data for binary variables were considered “negative”; however, no further explanation for these negative values was provided [47];
○ Evaluation of models’ performance: The predictive ability of an algorithm is assessed by the accuracy of the predicted outcomes in the test dataset [55]. Different measures are used to evaluate the predictive performance, for example, the area under the receiver operating characteristic curve (AUROC) [56], accuracy, sensitivity (i.e., recall), specificity, precision and F1 score [57].

To evaluate the predictive performance of their models, three studies [43,46,47] reported using AUROCs. In the study by Kanjilal et al., AUROC was poor for all antibiotics predictions [43], while in the study by Yelin et al., it ranged from acceptable to excellent for predicting susceptibility to amoxicillin-CA and ciprofloxacin, respectively [46], and in the study by Oonsivilai et al., it was excellent for all antibiotics susceptibility predictions [47]. On the other hand, two studies [44,45] reported using the recall and precision measures, with both measures ranging from 73–96%, which indicates high-performing models, in addition to the F1-score measure of 77% reported in [44], and the accuracy measure of 79% reported in [45] (Table 1).

○ Findings of included studies: All five studies [43,44,45,46,47] used ML models to predict their outcomes. They included both bedside and non-bedside predictors that were extracted from either EHRs, or information systems of healthcare services organisations, and thus the ML models included complete patients’ information for their development. Regarding the primary outcome, the study by Kanjilal et al. reported that their algorithm was able to make a recommendation for an antibiotic in 99% of the specimens, and chose ciprofloxacin or levofloxacin for 11% of the specimens, relative to 34% in the case of clinicians (a 67% reduction) [43].

On the other hand, the secondary outcome (prediction of inappropriate antibiotic prescriptions) was reported as the proportion of recommendations for inappropriate antibiotic therapy (i.e., second-line antibiotics) in the study by Kanjilal et al., whose algorithm’s recommendation resulted in an inappropriate antibiotic therapy (i.e., second-line antibiotics), in 10% of the specimens relative to 12% in case of clinicians (18% reduction) [43]. In the study by Lee et al., the authors reported success in predicting antibiotic prescription errors and that their algorithm was able to predict 145 prescription errors out of 179 predefined errors [44], while in the study by Beaudoin et al., pharmacists reviewed 374 prescriptions of piperacillin–tazobactam, of which 209 were defined as inappropriate [45]. The ML algorithm predicted 270 out of the 374 prescriptions as inappropriate, with a positive predictive value of 74%. Furthermore, in the study by Yelin et al., the authors reported that both algorithms used (unconstrained and constrained) were able to reduce the mismatched treatments as compared to prescribing by clinicians; the unconstrained resulted in a predicted mismatch of 5% (42% lower than the mismatch of 8.5% in case of clinicians), while the constrained resulted in a predicted mismatch of 6%, which is higher than the unconstrained models, however still lower than the 8.5% in case of clinicians [46]. In the study by Oonsivilai et al., the authors reported positive results in predicting susceptibility to antibiotics, which is used to guide antibiotic prescribing using their ML algorithms, and that was reflected by the AUROC values for the different ML models, rather than the results of the prediction models themselves [47]. In addition, the authors reported that when the performance of different ML algorithms was compared, the random forest algorithm outperformed the other ML algorithms in predicting susceptibility. (See Table 1). 

#### 3.1.3. Assessing the Risk of Bias

The assessment of the quality of the included studies in this review is shown in Table 2. Five studies [43,44,45,46,47] had a “Fair” rating, meeting 8 out of 14 criteria. Some of the criteria in this quality assessment tool [42] were not applicable to the studies included. For example, the third criterion (i.e., the participation rate for eligible persons), the fifth criterion (i.e., sample size justification), the eighth criterion (i.e., measurement of different levels or amounts of exposures), and the thirteenth criterion, (i.e., loss to follow-up). No study [43,44,45,46,47] reported any information regarding the 12th and 14th criteria (i.e., blinding of the outcome assessors and the measurement of the confounding variables).

## 4. Discussion

### 4.1. Summary of Main Findings

This review included five studies [43,44,45,46,47] to evaluate the use of artificial intelligence in improving antibiotic prescribing for human patients. All studies used supervised ML models as a means to improve prescribing, and all of them focused solely on antibiotics, and no other form of antimicrobial (i.e., antivirals or antifungals) was reported. One study [43] reported a relatively decreased second-line antibiotic (primary outcome), while all studies [43,44,45,46,47] reported the ability of their algorithms to predict inappropriate antibiotic prescriptions (secondary outcome). These findings align with what Fanelli et al. suggested, namely that ML contributes to increasing appropriate antibiotic therapies and minimising the risks of antibiotic resistance [58]. 

### 4.2. Using ML Models to Improve Antibiotic Prescribing

Developing an ML model in a healthcare context, such as the models in this review, developed to improve antibiotic prescribing, has to go through the following phases:

- Problem selection and definition;
- Data collection/curating datasets;
- ML development;
- Evaluation of ML models;
- Assessment of impacts;
- Deployment and monitoring [59].

Table 3 assesses the adherence of the studies included in this review to the prior mentioned six phases.

*Problem selection and definition*: The first step in developing ML models in a healthcare context is to carefully select and define the predictive task and make sure the data needed is available [59]. In addition, careful study design is necessary to generalise the models for future clinical use [60]. Furthermore, data variation [58], which leads to better model results [61,62,63], is achieved by preparing the dataset and choosing the predictors well [64]. All five studies [43,44,45,46,47] provided clear definitions for the problems they were modelling, and reported the size of the population included in their studies, which was not necessarily large, such as in the case of the study by Oonsivilai et al. (i.e., the number of patients included were 243) [47]. Furthermore, all studies [43,44,45,46,47] reported the predictors they used in their models, which were both bedside and non-bedside predictors. However, there was no justification for how or why these predictors were chosen or if there were any associations (which may result in multicollinearity) between them. 

*Data collection/curating datasets*: In this step, the datasets used in the model are constructed, and the development/validation sets (i.e., train/test splits) are generated [59]. None of the included studies [43,44,45,46,47] reported any information about their sample size calculation. Three studies [43,46,47] reported a train/test split for the data; however, no justification for this split was provided.

*ML development*: The choice of an ML model is based on several factors, such as size, type and complexity of data. Higher-performing ML models in problems relevant to ABR, such as support vector machines (SVM), random forests (RFs) and artificial neural networks (ANN), are less frequently used than simpler models, such as decision trees (DTs) and logistic regression (LR) [65]. All five studies [43,44,45,46,47] reported the ML models they used in their studies. In the study by Kanjilal et al., the authors reported using logistic regression based on interpretability and validation performance as compared to decision tree and random forest models [43]. However, they provided no justification for the three models they chose. And in the study by Lee et al., the authors reported that they collected the rules they used for their model, based on consultation with a clinician; however, no information was reported on further involvement of the clinician in the development of their model [44]. On the other hand, three studies [45,46,47] did not provide justification for their choices of the ML models they used, nor the suitability of these models for the nature or complexity of their data.

*Evaluation of ML model*: There are two categories for the evaluation measures of ML models, which are discrimination and calibration. Discrimination measures check the ability to distinguish or rank two classes, such as recall, precision, specificity and the area under the receiver operating characteristic curve (AUROC). On the other hand, the calibration measures evaluate how well the predicted probabilities match the actual probability, for example, the Hosmer–Lemeshow statistic. In addition, in healthcare research, subgroup analysis is used as an aspect of evaluation. Although this is not applicable in ML models, however, it can be conducted via the inclusion or exclusion of certain subgroups in an ML model [59]. All five studies [43,44,45,46,47] used discrimination measures to evaluate the performance of their models, and only two studies [43,45] reported age groups of included patients. In addition, in the study by Beaudoin et al., the authors reported that they used the results of a previous antimicrobial prescription surveillance system (APSS) used by three hospital pharmacists during the evaluation of their model’s performance [45].

*Assessment of Impact*: There are several challenges in using ML in a healthcare context, such as making sure a system based on an ML model would be user-friendly and reliable [59], in addition to considering automation bias [60]. None of the studies provided any information on whether their ML models were used or tested by prescribing clinicians or would be reliable tools to use in clinical practices to guide antibiotic prescribing. 

*Deployment and monitoring*: There are several factors to consider when implementing technologies based on ML models, such as hardware and software infrastructures, reliable internet, firewalls and ethical/ privacy/regulatory/legal frameworks [59]. None of the five studies [43,44,45,46,47] provided any information on deployment or monitoring. In addition, no information was provided about any ethical, privacy or legal requirements (i.e., approvals from health departments) for the models to be deployed as tools to guide antibiotic prescriptions in different healthcare settings. This implies that there is a gap between the current studies and their application as tools to guide antibiotic prescribing in the real world.

#### More on ML Models and Generalisation

In Section 3.1.2, methods to avoid data overfitting and improve generalisation were discussed. Moreover, there are two more factors that have an impact on a generalisation of an ML model: bias and robustness.

○ *Reproducing bias in ML models:* Bias can lead to the lack of generalisation in an ML model [66]. If an ML model is trained on a dataset that was generated on a biased process, the output of the model may also be biased (i.e., bias reproduction), which is a real challenge when using healthcare data sources, such as EHRs [67]. This type of bias is called “algorithmic bias” [66]. Other sources of bias in an ML model may be “data-driven” (for example, bias due to ethnicity or socioeconomic status) or “human”, in which the persons who develop the ML system reflect their personal biases [66]. Two of the included studies [43,46] reported biases in their data. In the study by Kanjilal et al. [43], the authors reported data bias, for most of their data was for Caucasian patients. And to minimise producing biased predictions, they used a national criterion adopted for uncomplicated urinary tract infections. The second bias reported was the prediction of non-susceptibility more often in environments in which the ABR prevalence is higher than what exists in the training data, and thus the authors have assessed the temporality by using longitudinal data and have confounded by indication [43]. In the study by Yelin et al. [46], the authors reported some aspects of the data they used that would introduce bias in their results, such as the later use of a purchased antibiotic (shall produce a bias towards higher odds ratio for purchases before infection), patients who used antibiotics that were not made via the information system the authors used to extract the data (shall produce a bias towards lower odds ratio for drug purchases), the UTIs that are empirically treated without culture (shall produce a bias towards measuring of more resistant samples), the elective culture testing for cultures made after failure of treatment (shall produce a bias towards measure of more resistant samples, in addition to a strong association of drug purchases and resistance), in addition to the dependence of elective cultures on demographics (shall produce associations between demographics and resistance). However, the authors did not report any means to avoid these biases, and they have reported that their models were still able to predict resistance well and for their recommendation algorithms to reduce the chances of antibiotic prescribing for a resistant infection [46]. However, this implied that there was still a chance to reproduce these biases in their predictions. On the other hand, the three remaining studies [44,45,47] did not report biases indicating the chance of bias reproducibility in their predictions;
○ *Robustness of ML models:* The robustness of an ML model is how well the model is trained to face adversarial examples [68]. It considers the sensitivity of the model (i.e., how the model’s output is sensitive to a change in its input) [69]. In addition, robustness has been used to derive generalisation in a supervised learning ML model [70]. All five studies [43,44,45,46,47] have used different performance measures, which help in understanding the sensitivity of their models and, thus, how robust they are. They reported measures such as recall (i.e., sensitivity), F1 score, AUROC, etc., which contribute to indicating how sensitive their models were to changes in inputs. In addition, in the study by Kanjilal et al. [43], the authors reported carrying out a sensitivity analysis with several false negative rates for each antibiotic to translate the output of their models into susceptibility phenotypes. And in the study by Beaudoin et al. [45], the authors reported that they selected a distance threshold (below which a prescription for piperacillin–tazobactam was classified as inappropriate) based on previous experimentations (i.e., sensitivity analysis).

### 4.3. ML Models Versus AB Prescribing Clinicians

The lack of information about the engagement of clinicians or their feedback in the development process of the ML models nor their usability and applicability in clinical settings may align with what the authors in the study by Waring et al. reported regarding the lack of ML expertise among healthcare workers [71]. In addition, this lack of engagement renders the use of ML in AB prescribing a theoretical exercise, rather than a promising tool for improving AB prescribing. Furthermore, the improvements in prescribing presented by the ML models compared to clinicians could be the result of the additional laboratory information, which was not available to the clinicians at the time of prescribing.

Limitations of this review lay in the difficulty of including relevant non-peer-reviewed publications in mediums, such as arXiv and the small number of included studies that made it unsuitable to use I2 statistics to assess their heterogeneity. In addition, not all of the included studies provided enough information about the reproducibility of bias, and avoiding overfitting in their models, nor their robustness, which makes the generalisability of these models inconclusive. And the lack of implementation in clinical practices makes it hard to understand the potential problems (i.e., hardware problems, software problems, training needs, etc.) towards successful adoption of these models. 

## 5. Conclusions

ML models may improve AB prescribing in different clinical settings; however, prescribing clinicians were not involved in the development process of the ML models nor in their evaluation of the ML models. Future research should consider a baseline ML model, developed with the same information that clinicians have at the time of prescribing, putting into consideration issues such as overfitting, bias reproduction, and robustness of their model to improve its generalisation. In addition, prescribing clinicians should be engaged in the development and deployment processes of ML models in clinical practices. Furthermore, it should explore the potential contribution of higher-performing ML models, such as support vector machines (SVMs) and artificial neural networks (ANNs), to improve AB prescribing.

## Figures and Tables

**Table 1:** Characteristics of ML models in included studies.

Title	Time Frame	Predicted (Outcome) Variable	Predictors’ Groups	Training/Test Sets	Machine Learning Models Used	Ways to Avoid Data Overfitting	Handling Missing Data	Evaluation of Models’ Performance	Results
A decision algorithm to promote outpatient antimicrobial stewardship for uncomplicated urinary tract infection [43].	10 years (1 January 2007–31 December 2016).	The proportion of recommendations for second-line antibiotics and the proportion of recommendations for inappropriate antibiotic therapy.	Labs, antibiotics, demographics, geographical, comorbidities and medical history of patients.	Training dataset (n = 10,053 patients, and thus 11,865 specimens) and test dataset (n = 3629 patients, and thus 3941 specimens).	Logistic regression, decision trees, random forest models.	Regularisation was used in the logistic regression model.	No information.	AUROCs for nitrofurantoin and TMP-SMX were poor (0.56 and 0.59). For ciprofloxacin and levofloxacin, the AUROCs were poor as well (0.64).	The ML model was able to make a recommendation for an antibiotic in 99% of the specimens, and chose ciprofloxacin or levofloxacin for 11% of the specimens, relative to 34% in the case of clinicians (a 67% reduction). Furthermore, the model’s recommendation resulted in an inappropriate antibiotic therapy (i.e., second-line antibiotics) in 10% of the specimens relative to 12% in the case of clinicians (18% reduction).
A hybrid method incorporating a rule-based approach and deep learning for prescription error prediction [44].	1 year (1 January–31 December 2018).	Antibiotic prescription errors.	Labs, medical history of patients and antibiotics.	No information.	An advanced rule-based deep neural network (ARDNN).	No information.	2.45% of height and weight missing data were predicted, and other empty data records were deleted. Data outliers were treated as missing values.	The performance was evaluated with a precision of 73%, recall of 81% and F1 score of 77%.	Out of 15,407 prescriptions by clinicians, there were 179 prescription errors. A validated prediction model for prescription errors correctly detected 145 prescription errors out of the 179 errors, implying a precision of 81%, recall of 73% and F1 score of 77%.
Evaluation of machine learning capability for a clinical decision support system to enhance antimicrobial stewardship programs [45].	11 months (2012 and 2013), where phase one was from 1 February to 30 November 2012, and phase two was from 18 November to 20 December 2013).	Inappropriate prescriptions of piperacillin–tazobactam.	Labs, demographics, geographical and vital signs.	No information.	A supervised learning module (temporal induction of classification models (TIM), which combines instance-based learning and rule induction).	The J-measure was used for measuring the improvement of a rule (i.e., the higher the information content of a rule reflected by the high J-measure, the higher the predictive accuracy of the model).	No information.	The overall system achieved a precision of 74%, recall of 96% and accuracy of 79%.	44 learned rules were extracted to identify inappropriate piperacillin–tazobactam prescriptions. When tested against the data set, they were able to identify inappropriate prescriptions with a precision of 66%, recall of 64% and accuracy of 71%.
Personal clinical history predicts antibiotic resistance to urinary tract infections [46].	10 years (1 July 2007–30 June 2017).	Mismatched treatment.	Labs, antibiotics, demographics, geographical, temporal and gender-related.	Training dataset: all data collected from 1 Jul 2007 to 30 Jun 2016; test dataset: all data collected from Jul 2016 to 30 Jun 2017.	Logistic regression and gradient-boosting decision trees (GBDTs).	The model performance on the test set was contrasted with the model performance on the training set to identify data overfitting.	Missing data for resistance measurements were defined as not available (N/A), and such samples were not used in the models.	AUROC was acceptable at 0.7 [amoxicillin-CA] to excellent at 0.83 [ciprofloxacin].	The unconstrained algorithm resulted in a predicted mismatch treatment of 5% (42% lower than the mismatch treatment of 8.5% in the case of clinicians’ prescriptions), and the constrained resulted in a predicted mismatch of 6%.
Using machine learning to guide targeted and locally tailored empiric antibiotic prescribing in a children’s hospital in Cambodia [47].	3 years (from February 2013 to January 2016).	Susceptibility to antibiotics.	Labs, antibiotics, demographics, temporal, socioeconomic conditions and medical history of patients.	The dataset was split 80% versus 20% for training and test datasets.	Logistic regression, decision trees, random forests, boosted decision trees, linear support vector machines (SVM), polynomial SVMs, radial SVMs and K-nearest neighbours.	Regularisation was used in the logistic regression model; however, no details about the rest of the ML models were used.	Missing data for the binary predictors were treated as being “negative”.	AUROC of the random forest method was excellent at 0.80 for ceftriaxone, acceptable at 0.74 for ampicillin and gentamicin and 0.71 for Gram-stain.	The random forest method had the best predictive performance in predicting susceptibility to antibiotics (such as ceftriaxone, ampicillin and gentamicin, and Gram-stain), which will be used to guide appropriate antibiotic therapy. In addition, the authors reported the AUROC values of different models rather than the ML models’ results.

**Table 2:** Assessment of Risk of Bias.

Criteria	A Decision Algorithm to Promote Outpatient Antimicrobial Stewardship for Uncomplicated Urinary Tract Infection [43]	Hybrid Method Incorporating a Rule-Based Approach and Deep Learning for Prescription Error Prediction [44]	Evaluation of a Machine Learning Capability for a Clinical Decision Support System to Enhance Antimicrobial Stewardship Programs [45]	Personal Clinical History Predicts Antibiotic Resistance of Urinary Tract Infections [46]	Using Machine Learning to Guide Targeted and Locally Tailored Empiric Antibiotic Prescribing in a Children’s Hospital in Cambodia [47]
1. Was the research question or objective in this paper clearly stated?	Yes	Yes	Yes	Yes	Yes
2. Was the study population clearly specified and defined?	Yes	Yes	Yes	Yes	Yes
3. Was the participation rate of eligible persons at least 50%?	NA	NA	NA	NA	NA
4. Were all the subjects selected or recruited from the same or similar populations (including the same time period)? Were inclusion and exclusion criteria for being in the study prespecified and applied uniformly to all participants?	Yes	Yes	Yes	Yes	Yes
5. Was a sample size justification, power description, or variance and effect estimates provided?	NA	NA	NA	NA	NA
6. For the analyses in this paper, were the exposure(s) of interest measured prior to the outcome(s) being measured?	Yes	Yes	Yes	Yes	Yes
7. Was the timeframe sufficient so that one could reasonably expect to see an association between exposure and outcome if it existed?	Yes	Yes	Yes	Yes	Yes
8. For exposures that can vary in amount or level, did the study examine different levels of the exposure as related to the outcome (e.g., categories of exposure or exposure measured as a continuous variable)?	NA	NA	NA	NA	NA
9. Were the exposure measures (independent variables) clearly defined, valid, reliable, and implemented consistently across all study participants?	Yes	Yes	Yes	Yes	Yes
10. Was the exposure(s) assessed more than once over time?	Yes	Yes	Yes	Yes	Yes
11. Were the outcome measures (dependent variables) clearly defined, valid, reliable, and implemented consistently across all study participants?	Yes	Yes	Yes	Yes	Yes
12. Were the outcome assessors blinded to the exposure status of participants?	NR	NR	NR	NR	NR
13. Was the loss to follow-up after baseline 20% or less?	NA	NA	NA	NA	NA
14. Were key potential confounding variables measured and adjusted statistically for their impact on the relationship between exposure(s) and outcome(s)?	NR	NR	NR	NR	NR
**Quality Rating**	**Fair (57.1%)**	**Fair (57.1%)**	**Fair (57.1%)**	**Fair (57.1%)**	**Fair (57.1%)**

Note: Quality would be rated poor if (0–4 out of 14 questions), fair if (5–10 out of 14 questions), and good if (11–14 out of 14 questions); NA: not applicable, NR: not reported.

**Table 3:** Adherence to the five phases of the development of a classifier.

Studies	Phase (1)	Phase (2)	Phase (3)	Phase (4)	Phase (5)	Phase (6)
	Problem Selection and Definition	Data Collection/Curating Datasets	ML Development	Evaluation of the ML Model	Assessment of Impact	Deployment and Monitoring
**A decision algorithm to promote outpatient antimicrobial stewardship for uncomplicated urinary tract infection** [43].	Yes	Yes, information about the dataset and training/test split is provided. However, no justification was provided for the train/test split.	Logistic regression was chosen based on a comparison with decision tree and random forest models; however, no justification was provided on why these three models were chosen.	Yes	No information	No information
**Hybrid Method Incorporating a Rule-Based Approach and Deep Learning for Prescription Error Prediction** [44].	Yes	Yes, information about the dataset is provided. However, no information was provided for the train/ test split.	No justification was provided for choosing the ML model; however, the authors reported that the rules they used with their model were based on consultation with a clinician.	Yes	No information	No information
**Evaluation of a machine learning capability for a clinical decision support system to enhance antimicrobial stewardship programs** [45].	Yes	Yes, information about the dataset is provided. However, no information was provided for the train/ test split.	No justification was provided for choosing the ML model.	Yes	No information	No information
**Personal clinical history predicts antibiotic resistance to urinary tract infections** [46].	Yes	Yes, information about the dataset and training/ test split is provided. However, no justification was provided for the train/test split.	No justification was provided for choosing the ML model.	Yes	No information	No information
**Using machine learning to guide targeted and locally tailored empiric antibiotic prescribing in a children’s hospital in Cambodia** [47].	Yes	Yes, information about the dataset and training/test split is provided. However, no justification was provided for the train/test split.	No justification was provided for choosing the ML model.	Yes	No information	No information

## Data Availability

Not applicable.

## Supplementary Materials

The following supporting information can be downloaded at: https://www.mdpi.com/article/10.3390/antibiotics12081293/s1, Table S1. Characteristics of the included studies; Table S2. Excluded studies (full-text screening); Table S3. Groups of predictors; Table S4. Excluded studies reasons (title & abstract screening).
