# Supplementary material for: Artificial Intelligence to Improve Antibiotic Prescribing: A Systematic Review
Source: Antibiotics (Basel). 2023 Aug 6;12(8):1293. doi: 10.3390/antibiotics12081293 (PMC10451640; doi:10.3390/antibiotics12081293)
Supplement: Supplementary file 1 [file antibiotics-12-01293-s001.zip › antibiotics-2468891-supplementary.pdf]

Supplementary Materials

Table S1. Characteristics of Included Studies

Publication Type	Country	Name of Publication Outlet	Authors	Year of Publication	Title	Aim of Study	Funding Source	Population	Total No. of Participants	Design	Setting	Start & End Dates	Outcomes	Outcome Definition	Key Conclusions
Journal Article	U.S.A	Antibiotics	**Kanjilal et al** [1]	2020	A decision algorithm to promote outpatient antimicrobial stewardship for uncomplicated urinary tract infection	Develop machine learning models to predict antibiotic susceptibility using electronic health record data and build a decision algorithm for recommending the narrowest possible antibiotic to which a specimen is susceptible.	This work was supported by a Massachusetts General Hospital–Massachusetts Institute of Technology Grand Challenges Award (S.K., M.O., S.B., and H.Z.), a Harvard Catalyst CMeRIT grant (S.K.), and a National Science Foundation CAREER award (S.B. and D.S.).	Female patients with uncomplicated urinary tract infections (UTIs).	*n* = 13,682 patients (*n* = 15,806 specimens) with uncomplicated UTI.	Retrospective study.	Hospital: Massachusetts General Hospital and the Brigham and Women's Hospital in Boston, MA.	1 Jan 2007–31 Dec 2016.	The proportion of recommendations for second-line antibiotics and the proportion of recommendations for inappropriate antibiotic therapy.	Inappropriate antibiotic therapy: "defined as the choice of a treatment to which a specimen is resistant".	The algorithm achieved a 67% reduction in the use of second-line antibiotics relative to clinicians. At the same time, it reduced inappropriate antibiotic therapy by 18% relative to clinicians. For specimens where clinicians chose a second-line drug but the algorithm chose a first-line drug, 92% (1066 of 1157) of decisions ended up being susceptible to the first-line drug. When clinicians chose an

															inappropriate first-line drug, the algorithm chose an appropriate first-line drug 47% (183 of 392) of the time.
Journal Article	South Korea	Drug Safety	**Lee et al** [2]	2022	Hybrid method incorporating a rule-based approach and deep learning for prescription error prediction	To develop a prescription error detection system.	This research was supported by the Korea Health Technology R&D Project through the Korea Health Industry Development Institute (KHIDI), funded by the Ministry of Health & Welfare, Republic of Korea (Grant number: HI17C2412) and by the National IT Industry Promotion Agency (NIPA) Grant funded by the Korean	Children.	15,281 patient-level observations of children.	Retrospective study.	Hospital: Konyang University Hospital (KYUH).	January 1 to December 31, 2018.	Antibiotic prescription errors.	An AB prescription error is defined based on 15 unit rules, such as dose caution, contraindication because of side effects, caution on weight, caution on usage, and contraindication for specific age groups.	A validated prediction model for prescription errors correctly detected 145 prescription errors out of 179 errors. Implying precision of 81.01%, recall of 72.9% and F1 score of 76.74%.

							government (MSIT) (No. A0105-20-1008, Development of AI-based detection service for prescription errors).								
Journal Article	Canada	Artificial Intelligence in Medicine	**Beaudoin et al** [3]	2016	Evaluation of a machine learning capability for a clinical decision support system to enhance antimicrobial stewardship programs	To evaluate a clinical decision support system for antimicrobial stewardship that can learn new rules supervised by user feedback.	This project was partially funded by the Fonds de recherche du Québec – Santé, the Fonds de recherche du Québec – Nature et technologies, the Natural Sciences and Engineering Research Council of Canada, and the Centre hospitalier universitaire de Sherbrooke.	In-patients who receive antimicrobials.	374 prescriptions, of which 209 were identified as inappropriate.	Prospective study.	Hospital: Centre Hospitalier Universitaire de Sherbrooke (CHUS), a 677-bed Canadian secondary- and tertiary-care hospital located at two distinct sites.	2-step process: first one 1 Feb 2012–30 Nov 2012, second one 18 Nov 2013 till 20 Dec 2013.	Inappropriate prescriptions of piperacillin–tazobactam.	Inappropriate antibiotic prescription: an antibiotic that has another safe alternative that is significantly less expensive but equally effective or an antibiotic to which the causative pathogen is resistant, making the treatment ineffective and endangering the life of the patient.	The learned rules were shown to extend the knowledge base of the baseline system by identifying pharmacist interventions that were missed by the baseline system. The learned rules identified prescribing practices that were not supported by local experts and were missing from its knowledge base.
Journal Article	Israel	Nature Medicine	**Yelin et al** [4]	2019	Personal clinical history predicts antibiotic	To analyse a large population of	N/A	Patients with UTIs	700,000 community-acquired urinary tract infections	Retrospective	Community and retirement home-	1 July 2007 and 30 June 2017	Mismatched treatment rate	Mismatched treatment rate: when the sample is resistant to the	Applying drug prescribing algorithms over a 1-year

					resistance to urinary tract infections	patients with UTIs to unravel predictive features of antibiotic resistance and test how these features can be combined to recommend optimal drugs for empirical treatment.			with over 5,000,000 individually resolved records of antibiotic purchases.		acquired UTI cultures collected by Maccabi Healthcare Services (MHS), Israel's second-largest health maintenance organisation.			prescribed antibiotic.	test period reduced the risk of mismatched treatment compared with the current standard of care (i.e., physicians' prescriptions).
Research Article	Cambodia	Wellcome Open Research	**Oonsivilai et al** [5]	2018	Using machine learning to guide targeted and locally-tailored empiric antibiotic prescribing in a children's	To propose a locally adapted decision support system for a Cambodian children's hospital by	This work was part of the Wellcome Trust-Mahidol University-Oxford Tropical Medicine Research Programme [106698/Z/14/Z]. BSC is	Children (less than 16 years old and with bloodstream infections).	243 patients.	Retrospective study.	Hospital: 100-bed children's hospital in North-West Cambodia.	Between February 2013 and January 2016.	Susceptibility to antibiotics.	The invasive pathogens' Gram stain and in vitro susceptibility to antibiotics.	Machine learning algorithms informed by relatively small amounts of patient-level data can be used to derive patient-specific predictions for empirical

					hospital in Cambodia	applying an array of machine learning algorithms to patient-level data. We evaluated the ability of the algorithms to predict whether the causative organisms are susceptible to i) ampicillin and gentamicin, ii) ceftriaxone, and iii) none of the above. We specifically focus on the value of using the	supported by the UK Medical Research Council and Department for International Development [MR/ K006924/1]. MY is supported by the Singapore National Medical Research Council Research Fellowship and National University Hospital [grant number NMRC/Fellowship/0051/ 2017]. The funders had no role in study design, data collection and analysis, decision to publish, or preparation								antibiotic therapy.

						predictive models to identify patients at high risk of being infected with organisms resistant to ceftriaxone, a third-generation cephalosporin, the most commonly prescribed empirical antibiotic in practice at the study site.	of the manuscript.								

Table S2. Excluded Studies (Full-text screening)

Publication Type	Name of Publication Outlet	Authors	Date	Study Title	Reason for Exclusion
Journal Article	Open Forum Infectious Diseases	J. C. H. Herigon, J.;Vernacchio, L.	2020	Derivation of novel phenotypes of outpatient paediatrician prescribing patterns	Different focus (i.e., focusing on creating prescribing phenotypes rather than improving prescribing).
Journal Article	International Journal of Antimicrobial Agents	S.-F. C. Huang, Jung-San;Sheu, Chau-Chyun;Liu, Yu-Ting;Lin, Ying-Chi	2016	An antibiotic decision-making tool for patients with pneumonia admitted to a medical intensive care unit	Different focus (i.e., focusing on multi-drug resistance).
Journal Article	International Journal of Medical Informatics	E. M. C. Nwanosike, Barbara R.;Merchant, Hamid A.;Hasan, Syed Shahzad	2022	Potential applications and performance of machine learning techniques and algorithms in clinical practice: A systematic review	Different focus (i.e., a systematic review of applications and performance of machine learning techniques and algorithms in clinical practice).
Journal Article	Artificial Intelligence in Medicine	G. C. Ochoa, Lee A.; Brownlee, Alexander E.; Hoyle, Andrew	2020	Multiobjective evolutionary design of antibiotic treatments	Different focus (i.e., no machine learning/ AI included).
Journal Article	Clinical Microbiology and Infection	N. R. Peiffer-Smadja, T. M.;Ahmad, R.;Buchard, A.;Georgiou, P.;Lescure, F. X.;Birgand, G.;Holmes, A. H.	2020	Machine learning for clinical decision support in infectious diseases: a narrative review of current applications	Antibiotic prescriptions aren't included.
Journal Article	Studies in health technology and informatics	W. C. Rödle, D.;Prokosch, H. U.;Kraus, S.	2020	Evaluation of Different Learning Algorithms of Neural Networks for Drug Dosing Recommendations in Pediatrics	Antibiotic prescriptions aren't included.
Journal Article	PloS one	J. N. van de Maat, Daan;Thompson, Matthew;Lakhanpaul, Monica;Moll, Henriette;Oostenbrink, Rianne	2019	Can clinical prediction models assess antibiotic needs in childhood pneumonia? A validation study in paediatric emergency care	Different focus (i.e., the study aimed at validation of prediction models for pneumonia).

Conference Paper	2018 13th IEEE Conference on Industrial Electronics and Applications (ICIEA)	Yifei Hu; Vincent C. S. Lee; Kenneth Tan	2018	Prediction of Clinicians' Treatment in Preterm Infants with Suspected Late-onset Sepsis - An ML Approach	Different focus (i.e., proposing and evaluating a general prediction model for clinicians' treatments for infants).
Journal Article	Artificial Intelligence in Medicine	Y. Y. Shen, Kaiqi;Chen, Daoyuan;Colloc, Joël;Yang, Min;Li, Yaliang;Lei, Kai	2018	An ontology-driven clinical decision support system (IDDAP) for infectious disease diagnosis and antibiotic prescription	Antibiotic prescriptions aren't included (note: although they are mentioned, however, there is no information about them).
Conference Paper	The 26th ACM SIGKDD Conference on Knowledge Discovery and Data Mining (KDD '20)	Soorajnath Boominathan, Michael Oberst, Helen Zhou, Sanjat Kanjilal, and David Sontag	2020	Treatment Policy Learning in Multiobjective Settings with Fully Observed Outcomes	Duplicate (Same data as 1491).

Table S3. groups of predictors

Group of predictors	Predictors Category	Predictors' Group	Study
1. Labs	Non-bedside	**Patient level:** **Microbiological**: urine cultures, blood cultures, bacterial species isolates, resistance profiles, and antibiotic susceptibility profiles **Laboratory**: potassium, glucose, blood urea nitrogen, creatinine, alanine transaminase, albumin, bilirubin, alkaline phosphatase, gamma-glutamyl transpeptidase, aspartate transaminase, prothrombin time, sodium and cystatin C, absolute neutrophil count (ANC), white blood cells count (WBC) and creatinine. **Population level**: Prevalence of resistance in urine specimens to an antibiotic in the 90 days preceding specimen submission.	Kanjilal et al [1], Lee et al [2,] Beaudoin et al [3], Yelin et al [4], Oonsivilai et al [5]
2. Antibiotics	Bedside	**Patient level**: Antibiotic exposures, prescriptions, wrong prescriptions, antibiotic purchases, antibiotics prior to admission (none, penicillin family, unknown). **Population level:** Hospital-wide antibiotic consumption.	Kanjilal et al [1], Lee et al [2], Yelin et al [4], Oonsivilai et al [5]
3. Demographics	Bedside	Gender, age, demographics, age-adjusted weight.	Kanjilal et al [1], Beaudoin et al [3], Yelin et al [4], Oonsivilai et al [5]
4. Geographical	Bedside	Hospital location of specimen collection, Patient location, retirement home residence.	Kanjilal et al [1], Beaudoin et al [3], Yelin et al [4]
5. Temporal	Bedside	Date of sampling, season of sampling, time from admission	Yelin et al [4], Oonsivilai et al [5]

6. Socioeconomic conditions	Bedside	Household size, water & sanitation data, and presence of domestic animals.	Oonsivilai et al [5]
7. Gender-related	Bedside	Pregnancy.	Yelin et al [4]
8. Comorbidities	Bedside	Comorbidities.	Kanjilal et al [1]
9. Vital signs	Bedside	Blood pressure, body mass index, respiratory rate, and temperature.	Beaudoin et al [3]
10. Medical history of patients	Bedside	Prior hospitalisation information, prior resistance, prior organism, complication during admission, Required ICU care/ ventilation, transfer from another hospital, admission differential diagnosis (sepsis, meningitis, lower respiratory tract infection/ pneumonia, upper respiratory tract infection, gastroenteritis, cellulitis, abscess, urinary tract infection), hospitalised in the last year (times), outpatient visits in last six months (times), treatment prior to current admission (pharmacy, nurse, traditional healer, received IV fluids, received medication), patient diagnostic information.	Kanjilal et al [1], [2], Oonsivilai et al [5]

1 Kanjilal S, Oberst M, Boominathan S, Zhou H, Hooper DC, Sontag D. A decision algorithm to promote outpatient antimicrobial stewardship for uncomplicated urinary tract infection. *Sci Transl Med* 2020; 12.

2 Lee S, Shin J, Kim HS, Lee MJ, Yoon JM, Lee S *et al*. Hybrid Method Incorporating a Rule-Based Approach and Deep Learning for Prescription Error Prediction. *Drug Saf* 2022; 45: 27-35.

3 Beaudoin M, Kabanza F, Nault V, Valiquette L. Evaluation of a machine learning capability for a clinical decision support system to enhance antimicrobial stewardship programs. *Artif Intell Med* 2016; 68: 29-36.

4 Yelin I, Snitser O, Novich G, Katz R, Tal O, Parizade M *et al*. Personal clinical history predicts antibiotic resistance of urinary tract infections. *Nat Med* 2019; 25: 1143-1152.

5 Oonsivilai M, Mo Y, Luangasanatip N, Lubell Y, Miliya T, Tan P *et al*. Using machine learning to guide targeted and locally-tailored empiric antibiotic prescribing in a children's hospital in Cambodia. *Wellcome Open Res* 2018; 3: 131.

Table S4. Excluded Studies Reasons (Title & abstract screening)

Reason	Number of studies
1. Manually identified duplicates	63 (1.7%)
2. AI not mentioned	29 (0.8%)
3. Wrong outcome	403 (10.9%)
4. Irrelevant study (i.e., one of the search terms appeared in the “keywords” of the study, however the study is completely of different focus)	812 (22.1%)
5. Wrong population + AI was not mentioned	3 (0.1%)
6. Wrong outcome + AI was not mentioned	2345 (63.7%)
7. Wrong population + wrong outcome	27 (0.7%)
**Total no. of excluded studies**	**3682 (100%)**
